# Dose-Dependent Effects of Allopurinol on Human Foreskin Fibroblast Cells and Human Umbilical Vein Endothelial Cells under Hypoxia

**DOI:** 10.1371/journal.pone.0123649

**Published:** 2015-04-01

**Authors:** Yu Sun, Jacob George, Sonia Rocha

**Affiliations:** 1 Centre for Gene Regulation and Expression, College of Life Sciences, University of Dundee, Dundee, United Kingdom; 2 Division of Medical Sciences, Ninewells Hospital and Medical School, Dundee, United Kingdom; University of Tennessee Health Science Center, UNITED STATES

## Abstract

Allopurinol, an inhibitor of xanthine oxidase, has been used in clinical trials of patients with cardiovascular and chronic kidney disease. These are two pathologies with extensive links to hypoxia and activation of the transcription factor hypoxia inducible factor (HIF) family. Here we analysed the effects of allopurinol treatment in two different cellular models, and their response to hypoxia. We explored the dose-dependent effect of allopurinol on Human Foreskin Fibroblasts (HFF) and Human Umbilical Vein Endothelial Cells (HUVEC) under hypoxia and normoxia. Under normoxia and hypoxia, high dose allopurinol reduced the accumulation of HIF-1α protein in HFF and HUVEC cells. Allopurinol had only marginal effects on HIF-1α mRNA level in both cellular systems. Interestingly, allopurinol effects over the HIF system were independent of prolyl-hydroxylase activity. Finally, allopurinol treatment reduced angiogenesis traits in HUVEC cells in an in vitro model. Taken together these results indicate that high doses of allopurinol inhibits the HIF system and pro-angiogenic traits in cells.

## Introduction

Allopurinol is an inhibitor of xanthine oxidase (XO) that has been in clinical use for the treatment of gout for over 50 years [1]. XO is an enzyme responsible for the successive oxidation of hypoxanthine and xanthine, resulting in the production of uric acid, the end-product of the purine catabolic pathway. A by-product of XO action in these reactions is the generation of reactive oxygen species (ROS) such as superoxide anion (O_2_^-^) and Hydrogen Peroxide (H_2_O_2_) from molecular oxygen [2, 3]. Therefore, the inhibition of XO by allopurinol has been hypothesised as a possible “oxygen-sparing” mechanism, which may be beneficial in patients with chronic hypoxia [4]. In addition to blocking uric acid production, inhibition of XO also leads to an increase in hypoxanthine and xanthine. While xanthine cannot be converted to purines, hypoxanthine can be converted to the purine ribonucleotides, adenosine and guanosine monophosphates [5]. Alteration of the levels of these ribotides maycause feedback inhibition of amidophosphoribosyl transferase, the first and rate-limiting enzyme of purine biosynthesis [6]. In addition, antioxidant or non-XO-related activities of allopurinol have been detected through dose-dependent experiments [7, 8].

The apparent beneficial role of XO inhibitors on chronic heart failure patients could be due to their ability to consistently improve endothelial dysfunction by reducing oxidative stress, rather than urate reduction per se [9, 10]. Clinical studies have shown that allopurinol can treat chronic heart failure, due to its inhibition of XO through improvements in peripheral vasodilator capacity and blood flow rather than a urate-lowering effect [11]. Other clinic trials show that allopurinol has a beneficial effect on Chronic Kidney Disease and inflammatory indices such as C-reactive protein, a downstream marker of post NF-κB activation [12]. The molecular mechanisms of allopurinol in cardiovascular disease and kidney disease are still not fully understood. The potential effects of allopurinol at the cellular level are various and possibly dose dependent.

Here, the possibility of allopurinol’s actions as an oxygen-sparing agent in hypoxia, was investigated. Human Umbilical Vein Endothelial Cells (HUVECs) and Human Foreskin Fibroblasts (HFFs) were used to investigate endothelial cells response to hypoxia and to help understand the mechanism of allopurinol *in vitro*. Analysis of HIF subunits and its targets were analysed in response to increasing doses of Allopurinol in normoxia and hypoxia. Finally, allopurinol effects on pro-angiogenic responses were analysed in a model of *in vitro* endothelial tube formation. Allopurinol produced dose dependent effects on HIF subunit levels and in some of its targets and resulted in reduced levels of pro-angiogenic features *in vitro*.

## Materials and Methods

### Cell culture

Human Umbilical Vein Endothelial Cells (HUVEC) (Gibco) were maintained in Medium 200 (Gibco) supplemented with Low Serum Growth Supplement (Gibco), 1% penicillin-streptomycin (Lonza), and 1% L-glutamine (Lonza) for no more than 8 passages. Human Forskin Fibroblasts (HFF) (ATCC cell collection) cells were maintained in DMEM (Dulbecco's modified Eagle's medium) (Lonza) supplemented with 10% (v/v) fetal bovine serum (Gibco), 1% penicillin-streptomycin (Lonza), and 1% L-glutamine (Lonza) for no more than 30 passages. Both of the cells were incubated at 37°C in a humidified environmental incubator supplemented with 5% CO_2_. Etoposide (Enzo) was used to induce apoptosis at 10 μM for 24 h. The cells used in this study are non-transformed cells and represent two different important cell types in diseases such as heart disease and cancer, namely fibroblast and endothelial cells. In addition, these cells would be expected to have different sensitivities to hypoxia exposure.

### Antibodies and Western blotting

Cells were lysed in a RIPA lysis buffer (50 mM Tris, pH 8.0, 150 mM NaCl, 1% NP-40, 0.1% SDS, 0.5% sodium deoxycholate, 250 mM Na_3_VO_4_, 10 mM NaF and protease inhibitor cocktail tablet). Western blotting was carried out using the following antibodies: anti-HIF-1α (R&D Systems #MAB1536, mouse monoclonal, 1:1000 dilution), anti-HIF-2α (Thermo Fisher Scientific #PA1-16510, rabbit polyclonal, 1:1000 dilution), anti-HIF-1β (Cell Signaling Technology #5537, rabbit polyclonal, 1:1000 dilution), anti-GLUT1 (glucose transporter 1) (Thermo Fisher Scientific #RB-9052-P, rabbit polyclonal, 1:1000 dilution), anti-Glut3 (AnaSpec #53520, rabbit polyclonal, 1:1000 dilution), anti-β-actin (Sigma #A5441, rabbit polyclonal, 1:5000 dilution), anti-cleaved PARP [poly(ADP-ribose) polymerase] (Cell Signaling Technology #9541, rabbit polyclonal, 1:1000 dilution), anti-PHD2 (Bethyl #A300-322A, rabbit polyclonal, 1:1000 dilution), anti-PHD3 (Bethyl #A300-327A, rabbit polyclonal, 1:1000 dilution), anti-NDRG1 (Cell Signaling Technology #9485, rabbit monoclonal 1:1000 dilution), anti-Cited-2 (Santa Cruz Biotechnology #sc-25375, rabbit polyclonal 1:500 dilution) and hydroxylated P564-HIF-1α (Cell Signaling Technology #3434, rabbit monoclonal 1:1000 dilution). All images are representative of a minimum of three independent experiments, ranging from 3–6.

### Western blot quantification

Western blots were analysed using ImageJ software to determine band intensity, normalised to the loading control signal. Control conditions bands were set at 1, and treatments with Allopurinol compared to control. Data is presented as the mean and standard deviation of a minimum of three independent experiments.

### Hypoxia inductions and MG132 treatment

Cells were incubated at 1% oxygen in an INVIVO_2_ 300 hypoxia workstation (Ruskinn). Cell extracts for protein and RNA were performed inside the workstation to avoid re-oxygenation. For hypoxia-mimetic conditions, cells were treated with 200 μM desferrioxamine (DFX, Sigma) for 16 h prior to harvest using passive lysis buffer (Promega). MG132 (Merck Biosciences) at 20 μM was added 3 h prior to cell harvesting.

### qPCR and PCR sequences

Total RNA extraction was performed using PeqGOLD Total RNA Kit (Peqlab) according to the manufacturer's directions. RNA was converted to cDNA using QuantiTect Reverse Transcription Kit (Qiagen). Relative quantitative RT-PCR assay was performed using cDNA templates amplified using specific primer sets and the Brilliant II SYBR Green qPCR Low ROX Master Mix (Stratagene) according to the manufacturer's instructions. Amplification and detection were performed using a Stratagene Mx3005P detection system. During amplification, a detector monitored real-time PCR amplification by quantitative analysis of the fluorescence emissions. Sample values obtained with specific primer sets were normalized to β-actin primer set values. The PCR primer sequences used were as described previously [13]. Data analysis was provided by the MxPro software (Stratagene/Agilent) based on the ∂∂CT method. All data presented in the mean of a minimum of three independent experiments performed in duplicate.

### Endothelial Tube formation assay

Angiogenesis slides (Ibidi) were coated with 10 μL Growth Factor Reduced Matrigel (Becton Dickinson) per well and incubated for 30 min at 37°C. HUVEC were detached by trypsin and adjusted to 100000 cells per ml with allopurinol at 0, 10, 100, and 1000 μg/ml. Cell suspension (50 μL per well) was seeded into each well. After 24 h, cells were photographed and analysed by ImageJ. All experiments were carried out in triplicate.

### Statistical analysis

ANOVA and Student’s t-tests were performed to determine differences among groups and p values were calculated. Data were expressed as mean values ± Standard Deviation of at least three independent experiments (SigmaPlot). Statistical significance was defined as * *P*<0.050, ** *P*<0.01, *** *P*<0.001.

## Results

### Characterisation of the response in HFF and HUVEC cells under hypoxia

**Fig 1:**
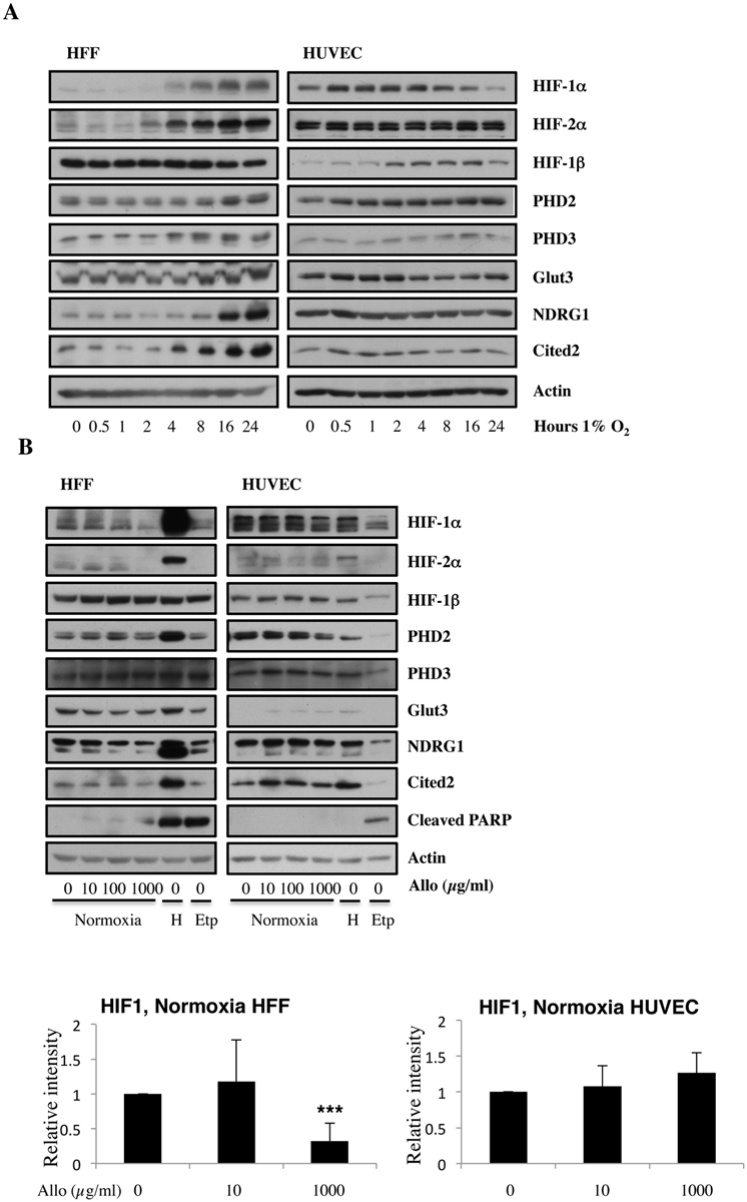
Increasing doses of Allopurinol reduce HIF-1α levels in normoxic HFFs and HUVEC cells. A. Characterisation of HFF and HUVEC response to hypoxia. Cells were exposed to hypoxia (1% oxygen) for the indicated periods of time. At the end of incubation, protein levels were determined in whole cell extracts by immunoblot analysis using the depicted antibodies. B. Cells were treated with Allopurinol at 10, 100 and 1000 μg/ml for 17 hours. Then the cells were lysed for assessment of the indicated protein levels. Cells were treated with Etoposide (Etop) for 24 hours under normoxia. H-cells exposed to 1% O2 for 16 hours. HIF-1α levels were quantified using ImageJ software and graph depicts mean and standard deviation of a minimum of three independent experiments. Anova t-test was performed and p values calculated as follows: *p<0.05; **p<0.01; ***p<0.001.

Since hypoxia is a major feature of cardiovascular disease [14], we aimed to characterise the hypoxia response in two different cellular backgrounds. Hypoxia-inducible factors and in particular, the oxygen labile 1-α subunit (HIF-1α) are principle regulators of cellular hypoxia adaptation [15]. However, different cell types may have different kinetics of the hypoxia response. To understand how two different cellular systems respond to hypoxia we performed a time course analysis for protein expression of HIF subunits and a selection of its targets in HFF and HUVEC in hypoxia. HFF and HUVEC were incubated in 1% oxygen from 0 to 24 hours. Both HFF and HUVEC responded to hypoxia by inducing HIF-1α protein levels (Fig 1A). While HUVEC cells start to reduce HIF-1α levels around 2 hours, in HFF cells this was not observed. For both types of cells, the accumulation of HIF-2α protein was relatively slower than the accumulation of HIF-1α, as it has been seen for cancer cells [16]. HIF-1β protein levels remained relatively constant, as HIF-1β is not sensitive to oxygen availability (Fig 1A).

Under normoxic conditions, the degradation of HIF-α is triggered by the hydroxylation of specific proline residues by prolyl hydroxylases 1, 2 and 3 (PHD1, 2 and 3), which create a binding site for the Von Hippel-Lindau tumour-suppressor protein [17]. Since PHDs have an absolute requirement for molecular oxygen, under hypoxia PHDs activity is reduced, and the degradation of HIF-α is blocked. This in turn, capacitates HIF-α transcription to activate hypoxia-responsive target genes [18]. Several HIF-dependent targets were analysed in HFF and HUVEC cells following hypoxia (Fig 1A). Both PHD2 and PHD3 protein levels were increased after prolonged hypoxia. This increase is likely responsible for the reduction in HIF-1α protein levels in HUVEC cells after 8 hours (Fig 1A). In addition, Glut3 was also induced in both cell lines, although the kinetics of activation was different for HFF and HUVEC cells.

Finally, the levels of Cited2 and NDRG1 proteins were also analysed. These genes can be activated by both HIF-1α and HIF-2α or HIF-2α alone [19, 20]. Interestingly, these genes were only induced following hypoxia in the HFF cells but not in HUVEC cells (Fig 1A). This analysis revealed the similarities and differences between the two cellular backgrounds, and highlighted the differential activation of the HIF system under hypoxia.

### Allopurinol alters normoxic HIF responses in HFF but not HUVEC cells

To determine if allopurinol can regulate basal or normoxic HIF levels, we performed a dose dependent response analysis in both HFF and HUVEC cells. Cells were treated with increasing amounts of allopurinol for 17 hours under normoxia conditions. To observe HIF-α under normoxic conditions, we exposed our western blots much longer compared to hypoxic conditions, as it can be seen when comparing Fig 1A with Fig 1B. Allopurinol treatment resulted in dose-dependent effects in both of the cell types analysed. In HFF, allopurinol treatment at 1000 μg/ml substantially reduced HIF-1α and HIF-2α protein expression, while HIF-1β expression was mostly unaffected by allopurinol treatment (Fig 1B).

To determine if the reduction of basal HIF levels was altering the expression of HIF-dependent target genes, we analysed the levels of several HIF targets at the protein level, following a dose dependent treatment with allopurinol (Fig 1B). The analyses of the relationship between HIF targets at protein levels and dose-dependent increases of alloprinol revealed a differential response in HFF and HUVEC cells. While in HFF, treatment with a higher dose of allopurinol resulted in reduced levels of the majority of HIF dependent target genes analysed, in HUVEC cells we could not detect any alteration in the proteins we have analysed. Analysis of the levels of cleaved PARP, a marker for apoptosis, indicated that allopurinol treatment was not inducing apoptosis in either of the cellular backgrounds analysed (Fig 1B).

This analysis revealed that HFF and HUVEC cells not only have different kinetics in their response to hypoxia but also have different sensitivity to allopurinol treatment under normoxic conditions.

### Allopurinol treatment reduces hypoxic HIF-1α levels in HFF and HUVEC cells but only partially affects HIF responses

**Fig 2:**
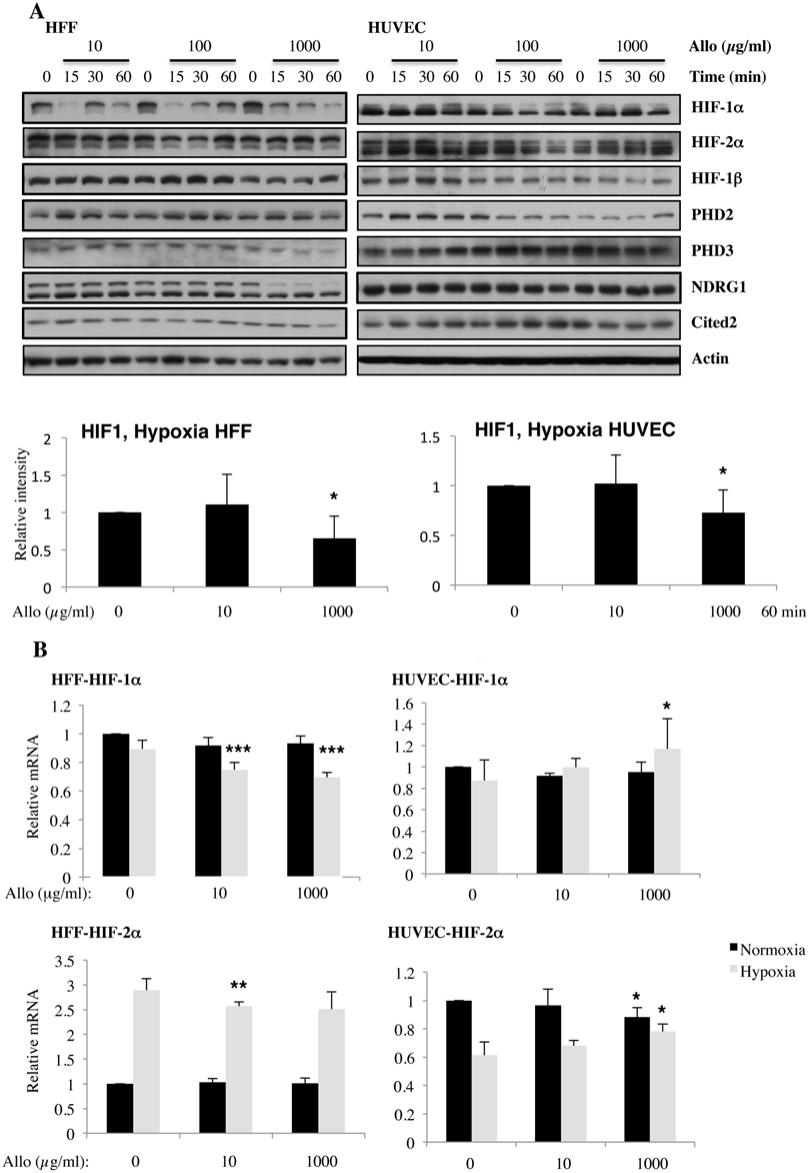
Increasing doses of Allopurinol reduce HIF-1α levels in hypoxic HFFs, without changing HIF mRNA levels but have reduced effect in HUVEC cells. A. Cells were pre-treated with Allopurinol at 10, 100 and 1000 μg/ml for 5, 30 and 60 minutes then were incubated in 1% oxygen for 16 hours. The cells were lysed for assessment of the indicated proteins. HIF-1α levels were quantified using ImageJ software and graph depicts mean and standard deviation of a minimum of three independent experiments. Anova t-test was performed and p values calculated as follows: *p<0.05; **p<0.01; ***p<0.001. B. HIF-1α and HIF-2α mRNA levels were analysed by quantitative PCR in HFF and HUVEC cells. Graph depicts the mean and standard deviation of a minimum of three independent experiments performed in duplicate. Anova t-test was performed and p values calculated as follows: *p<0.05; **p<0.01; ***p<0.001.

Since HIF transcription factors are mostly active in hypoxia, we next determined how allopurinol affects the HIF response in hypoxia. HIF-1α levels were reduced with treatment with high doses of allopurinol in both cell types (Fig 2A). HIF-2α and HIF-1β levels were not altered significantly by treatment with allopurinol in hypoxia, opposite to the effects seen in normoxia. In addition, when HIF-dependent targets were analysed under these conditions, only high doses of allopurinol, resulted in reduced HIF targets expression in hypoxia, but once again only in HFF cells but not in HUVECs (Fig 2A). These results again, support the hypothesis that HUVEC cells are less sensitive to allopurinol treatment than HFFs when HIF-dependent responses are analysed.

To determine if allopurinol treatment affects HIF levels by interfering with their mRNA, we next measured HIF-1α and HIF-2α transcript levels by qPCR. Our analysis revealed that allopurinol has no significant effect in the levels of HIF-1α or HIF-2α mRNA in normoxia in both cell lines tested (Fig 2B). In hypoxia, we could only detect statistical differences in the levels of HIF-1α and HIF-2α in HFF cells following allopurinol treatment, while HUVEC cells were only altered with higher doses (Fig 2B). Of note, although statistically significant, the mRNA changes observed following treatment with allopurinol are only minimal, and as such, we would not expect these changes to be biologically significant, as it is observed in the protein level.

### Allopurinol alters HIF levels independently of PHD function

**Fig 3:**
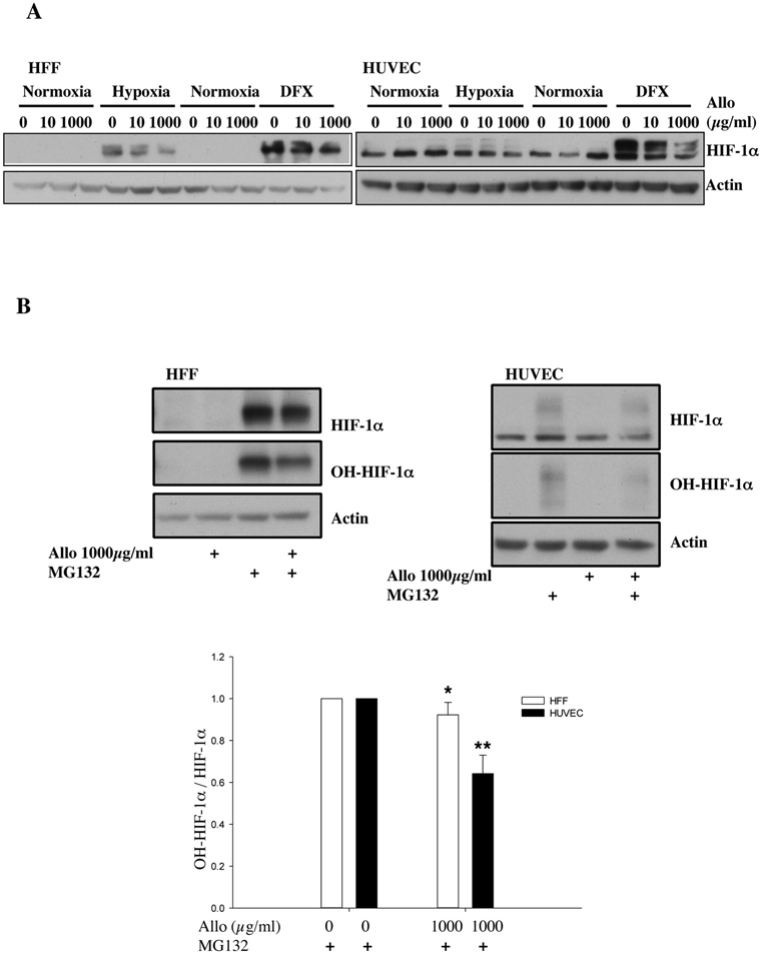
Allopurinol reduces HIF-1α levels independent of PHD function. A. Cells were pre-treated with Allopurinol at 10 and 1000 μg/ml for 60 minutes and then were incubated in 1% oxygen or treated with 200 μM DFX for 16 hours. Whole cell lysates were analysed by immunoblot using the indicated antibodies. B. Cells were pre-treated with Allopurinol at 1000 μg/ml for 60 minutes and then were incubated with 20μM MG132 for 3 hours. Whole cell lysates were analysed by immunoblot using the indicated antibodies. HIF-1α levels were quantified using ImageJ software and graph depicts mean and standard deviation of a minimum of three independent experiments. Anova t-test was performed and p values calculated as follows: *p<0.05; **p<0.01; ***p<0.001.

To address the involvement of PHDs in the effects seen after allopurinol treatment, we used desferrioxamine (DFX), an iron chelator, to inhibit PHD activity and thus stop the PHD dependent HIF-1α degradation pathway (Fig 3A). Both HFF and HUVEC cells showed a robust up regulation of HIF-1α protein following DFX treatment (Fig 3A). Interestingly, allopurinol treatment resulted in a dose-dependent reduction of HIF-1α protein levels in both cell lines. These results suggest that in HUVEC cells, allopurinol treatment will also result in reduced HIF-1α levels, but that these cells require a more dramatic reduction in oxygen to activate the HIF pathway. In addition, it demonstrates that allopurinol induced HIF-1μ reduction is independent of PHD activity.

To fully demonstrate that allopurinol is not increasing the PHD-dependent degradation pathway, we used MG132, a cell-permeable proteasome inhibitor in the absence or presence of allopurinol treatment (Fig 3B). As expected, MG132 treatment resulted in stabilisation of HIF-1α protein levels in both cell types (Fig 3B). However, in the presence of allopurinol, HIF-1α levels were not fully restored by MG132 treatment. In addition, we also observed reduced levels of hydroxylated HIF-1α, supporting our findings that PHD levels are reduced by allopurinol treatment (Fig 3B). Taken together, these data indicate that allopurinol treatment alters HIF levels via a PHD-independent pathway.

### Allopurinol reduces angiogenesis traits of HUVEC cells

**Fig 4:**
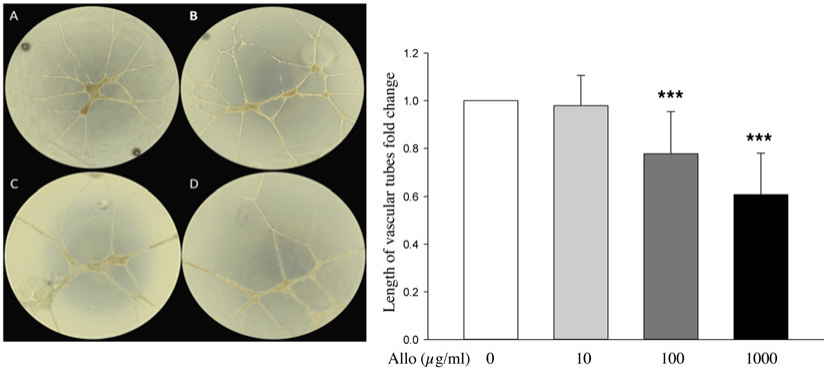
Allopurinol alters angiogenic traits of HUVEC in an *in vitro* endothelial tube model. Cells were treated with Allopurinol at 0, 10, 100, 1000 μg/ml, and analysed at 24 hours. A, control group without Allopurinol. B, 10 μg/ml Allopurinol. C, 100 μg/ml Allopurinol. D, 1000 μg/ml Allopurinol. Graph depicts mean and standard deviation of tube length measures in a minimum of three independent experiments performed in triplicate. Anova t-test was performed and p values calculated as follows: *p<0.05; **p<0.01; ***p<0.001.

Endothelial cells are important in blood vessels and, as such, can be used as an *in vitro* model of angiogenesis [21]. To determine if allopurinol alters HUVEC cells ability to produce endothelial tubes, we treated these cells with increasing concentrations of allopurinol and measured characteristics of this model (Fig 4). While the low dose of allopurinol, had no effect on total tube length, increasing doses of allopurinol resulted in a progressive reduction in the tube length (Fig 4). These results suggest that allopurinol can alter angiogenic potential of endothelial cells.

## Discussion

Here, we investigated two different cellular models and how they respond to hypoxia and treatment with allopurinol. Endothelial cells have a shorter period of hypoxia adaptation than HFF cells, indicated by the timing of HIF-1α activation and degradation. This is not surprising as endothelial cells are exposed to much higher oxygen levels than most tissues and as such need a quicker adaptation period to hypoxia, in order to survive [22]. We found that overall HFF cells are more sensitive to allopurinol treatment both in normoxia and hypoxia, when compared with HUVEC cells. Nevertheless, HUVEC cells were affected by higher doses of allopurinol when analysed for the ability to produce endothelial tubes in vitro. Furthermore, we observed that allopurinol effects over the HIF system were independent of PHD function and HIF subunit mRNA levels. Interestingly, the reduction observed in HIF-1α levels following allopurinol treatment was not reversed when the proteasome was inhibited. This suggests that allopurinol alters either HIF protein translation rates or proteasomal-independent degradation pathways.

Allopurinol was recently shown to control mTOR activity [23], a master regulator of protein synthesis in the cell [24]. mTOR is a known regulator of HIF-1α translation [25, 26], and as such could provide an explanation for our finding of reduced HIF-1α levels in the presence of allopurinol, even with the DFX treatment, that completely inhibits PHD function in cells. Further research, analysing mTOR activity following treatment with allopurinol in these cells, as well as activation marks for AMPK, a master regulator of mTOR [27], would be required to answer this particular question.

While mTOR regulation by allopurinol is a possible and viable explanation for the reduction observed in HIF-1α, alternatives mechanisms could also be involved. Recently, proteasomal-independent degradation pathways have been described for HIF-1α, including lysosomal and autophagy [28]. While there is some evidence that allopurinol can prevent lysosome enzyme release in disease conditions, the precise analysis of lysosomal degradation pathways in the presence of this drug has not yet been accessed. Similarly, the effects of allopurinol of the process of autophagy have not been investigated yet, making any assumption concerning the involvement of these pathways in the effects observed in this study difficult to document.

Endothelial cells are an essential component of blood vessels and their functional integrity [29]. One interesting finding from this study, was the reduction in angiogenic traits of HUVECs when treated with higher doses of allopurinol. Neo-angiogenesis is a key feature of most solid tumours, and it thought to contribute to the tumour growth and metastatic potential, due to the leakiness of the vessels made [30]. Interestingly, allopurinol has been used in cancer, as a way to block common side effects of routinely used chemotherapies [31, 32]. However, no report exists on the effects of allopurinol in the process of angiogenesis or vessel normalisation. This would be an interesting aspect to investigate, given our results with HUVEC cells, in particular in highly angiogenesis dependent cancers such as glioblastoma or breast cancer. Allopurinol could thus be a substitute for anti-angiogenic therapy that in recent years has proven to be relatively unsuccessful, with the cancer finding alternative routes to induce the process [33]. This may also lend support to the hypothesis that allopurinol may have a sparing effect on molecular oxygen as seen in clinical trials of patients with coronary artery disease where allopurinol significantly prolonged time to the development of electrocardiographic ischemic changes on an exercise tolerance test [4].

Overall, allopurinol did not change cell viability, despite changing HIF levels in hypoxia. It is possible that the HIF levels that remain are sufficient to maintain viability of cells. It would be interesting to test if under hypoxic conditions, in the presence of allopurinol, additional chemotherapeutic or even radiotherapy would have a higher therapeutic index. Additional work is thus necessary to answer these questions.
